# Supplementary material for: An in vitro model for vitamin A transport across the human blood-brain barrier
Source: bioRxiv. 2023 Apr 12:2023.04.11.536348. Preprint. [Version 1] doi: 10.1101/2023.04.11.536348 (PMC10120720; doi:10.1101/2023.04.11.536348)
Supplement: Supplement 1 [file NIHPP2023.04.11.536348v1-supplement-1.pdf]

bioRxiv preprint doi: https://doi.org/10.1101/2023.04.11.536348; this version posted April 12, 2023. The copyright holder for this preprint (which was not certified by peer review) is the author/funder, who has granted bioRxiv a license to display the preprint in perpetuity. It is made available under aCC-BY 4.0 International license.

SUPPORTING INFORMATION

An in vitro model for vitamin A transport across the human blood-brain barrier

Chandler B. Est[2] and Regina M. Murphy[1]

Department of Chemical and Biological Engineering, University of Wisconsin – Madison

1415 Engineering Dr., Madison, WI 53706

[1] Corresponding author. *E-mail address:* regina.murphy@wisc.edu

[2] Current address: Division of Endocrinology, Metabolism and Lipid Research, Washington University School of Medicine, 660 South Euclid Avenue, St. Louis, MO, 63110

bioRxiv preprint doi: https://doi.org/10.1101/2023.04.11.536348; this version posted April 12, 2023. The copyright holder for this preprint (which was not certified by peer review) is the author/funder, who has granted bioRxiv a license to display the preprint in perpetuity. It is made available under aCC-BY 4.0 International license.

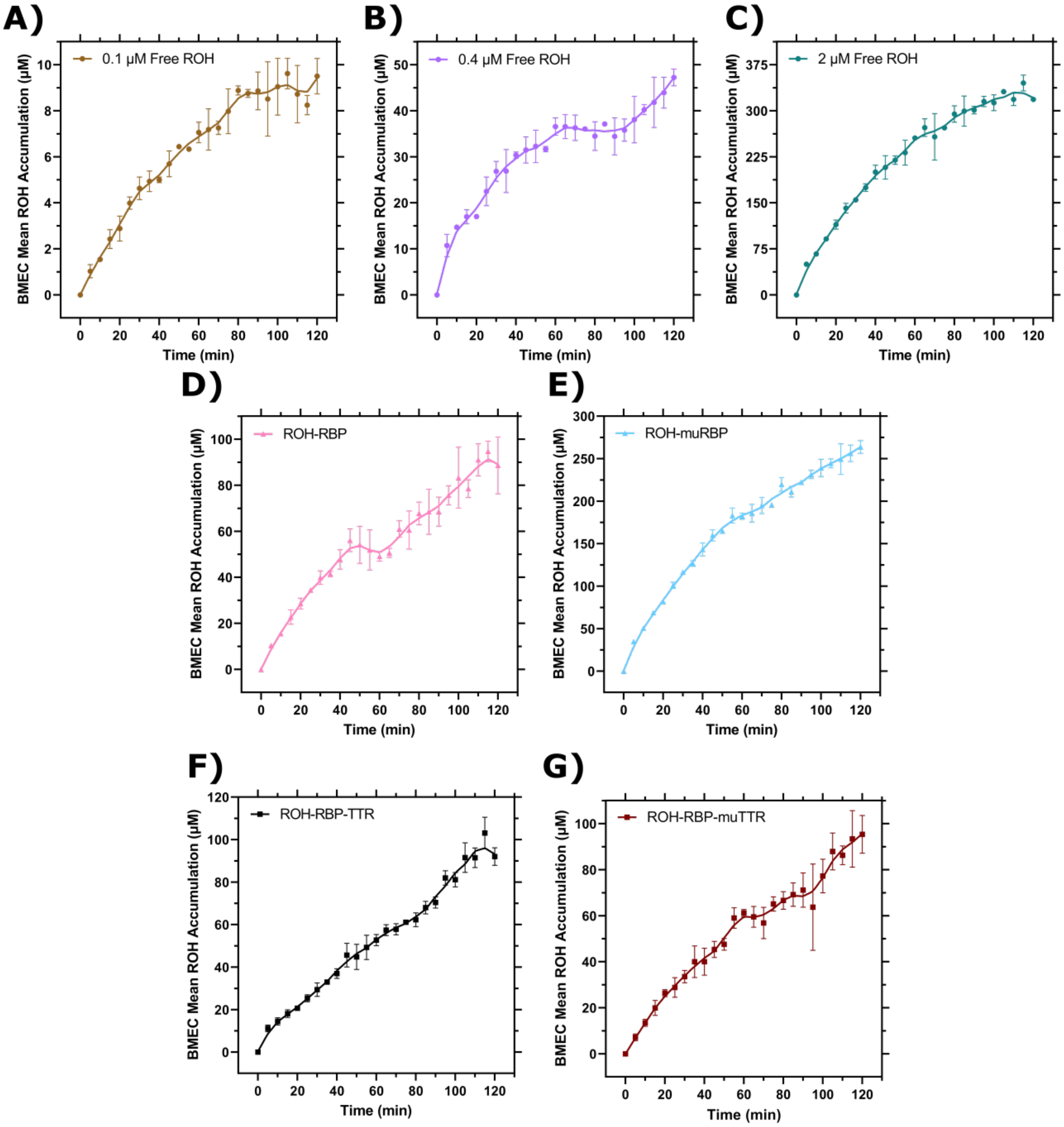

Figure S1: Individual BMEC ROH accumulation curves. Measured DPM values were converted to cellular concentrations using the specific activity of ^{3}H-ROH, the ^{3}H-ROH: unlabeled ROH ratio (1:20) and the calculated cell volume. Error bars represent the standard deviation of three biological replicates. Data are fit by a smoothed 10 point LOWESS function in order to better view the kinetic regimes. **A)** 0.1μM Free ROH; **B)** 0.4μM Free ROH; **C)** 2 μM Free ROH; **D)** ROH-RBP; **E)** ROH-muRBP; **F)** ROH-RBP-TTR; **G)** ROH-RBP-muTTR.

bioRxiv preprint doi: https://doi.org/10.1101/2023.04.11.536348; this version posted April 12, 2023. The copyright holder for this preprint (which was not certified by peer review) is the author/funder, who has granted bioRxiv a license to display the preprint in perpetuity. It is made available under aCC-BY 4.0 International license.

Table S1: Mass balances for ^{3}H-ROH

Sample	Replicate	Apical Counts x 10^{-3} (t0)	Apical Counts x 10^{-3} (t60)	Basolateral Counts x 10^{-3} (t60)	Lysate Counts x 10^{-3} (t60)	Missing Counts x 10^{-3} (t60)	% Missing
0.1 µM Free ROH	1	227.22	218.21	4.05	7.33	(2.38)	-1.0%
	2	235.00	218.08	4.77	7.26	4.89	2.1%
	3	238.90	218.61	4.68	8.23	7.37	3.1%
	4	233.22	219.94	4.98	8.83	(0.52)	-0.2%
0.4 µM Free ROH	1	326.57	295.66	7.81	13.70	9.40	2.9%
	2	326.87	291.44	8.98	13.43	13.02	4.0%
	3	327.87	292.72	9.28	13.44	12.43	3.8%
	4	333.21	300.92	10.26	15.28	6.75	2.0%
2 µM Free ROH	1	1,552.98	1,171.46	58.02	164.43	159.07	10.2%
	2	1,537.16	1,161.60	57.20	171.93	146.43	9.5%
	3	1,545.32	1,206.34	63.14	169.63	106.21	6.9%
	4	1,547.17	1,173.19	65.65	184.56	123.77	8.0%
ROH-RBP	1	1,799.84	1,675.54	60.68	49.51	14.11	0.8%
	2	1,840.65	1,688.63	62.44	53.72	35.85	1.9%
	3	1,848.14	1,681.03	62.71	54.53	49.87	2.7%
	4	1,819.80	1,680.83	66.05	63.90	9.01	0.5%
ROH-muRBP	1	1,534.63	1,427.66	59.51	67.42	(19.96)	-1.3%
	2	1,594.58	1,436.75	59.61	70.31	27.91	1.8%
	3	1,596.38	1,457.58	62.00	83.02	(6.22)	-0.4%
	4	1,555.79	1,441.91	62.05	74.77	(22.95)	-1.5%
ROH-RBP-TTR[1]	1	843.25	755.65	38.36	23.94	25.29	3.0%
	2	842.47	798.55	36.95	27.37	(20.40)	-2.4%
	3	826.47	778.79	36.47	23.65	(12.45)	-1.5%
	4	842.34	795.80	36.42	23.15	(13.03)	-1.5%
ROH-RBP-muTTR[1]	1	717.36	681.12	35.87	16.56	(16.19)	-2.3%
	2	724.06	679.06	36.40	15.12	(6.52)	-0.9%
	3	736.88	683.53	37.13	15.02	1.20	0.2%
	4	731.62	679.60	37.71	15.34	(1.03)	-0.1%

[1] TTR and muTTR samples were prepared with a target ^{3}H-ROH : unlabeled-ROH ratio of 1:40 in order to reduce the ethanol concentration required for 2X ^{3}H-ROH-RBP stocks. Precipitation of RBP was observed in 2X stocks prepared for use at 1:20 ratios. This problem only affected TTR and muTTR preparations because ROH-RBP and ROH-muRBP stocks could be prepared at 1X.

bioRxiv preprint doi: https://doi.org/10.1101/2023.04.11.536348; this version posted April 12, 2023. The copyright holder for this preprint (which was not certified by peer review) is the author/funder, who has granted bioRxiv a license to display the preprint in perpetuity. It is made available under aCC-BY 4.0 International license.

Table S2: Mass balances for ^{14}C-sucrose

Sample	Replicate	Apical Counts x 10^{-3} (t0)	Apical Counts x 10^{-3} (t60)	Basolateral Counts x 10^{-3} (t60)	Lysate Counts x 10^{-3} (t60)	Missing Counts x 10^{-3} (t60)	% Missing
0.1 µM Free ROH	1	590.78	603.22	2.48	6.70	(21.62)	-3.7%
	2	608.26	602.21	2.28	5.86	(2.09)	-0.3%
	3	612.02	600.11	2.75	8.73	0.44	0.1%
	4	604.47	599.49	2.75	11.88	(9.65)	-1.6%
0.4 µM Free ROH	1	659.44	657.39	2.77	5.84	(6.56)	-1.0%
	2	665.84	647.03	4.25	4.32	10.24	1.5%
	3	666.92	645.73	3.21	3.94	14.04	2.1%
	4	666.63	663.05	3.63	5.43	(5.47)	-0.8%
2 µM Free ROH	1	649.77	631.68	3.35	4.39	10.35	1.6%
	2	645.77	633.23	3.33	5.15	4.05	0.6%
	3	646.95	634.52	3.15	4.55	4.73	0.7%
	4	648.13	628.08	3.23	5.64	11.19	1.7%
ROH-RBP	1	663.97	678.58	4.36	5.13	(24.11)	-3.6%
	2	663.64	668.09	5.16	6.18	(15.79)	-2.4%
	3	670.71	667.15	4.55	5.38	(6.37)	-0.9%
	4	662.58	680.76	4.97	9.01	(32.17)	-4.9%
ROH-muRBP	1	639.32	664.00	5.06	5.32	(35.06)	-5.5%
	2	651.86	660.14	4.83	5.11	(18.21)	-2.8%
	3	654.17	662.85	5.01	5.14	(18.83)	-2.9%
	4	642.95	662.28	5.57	5.70	(30.60)	-4.8%
ROH-RBP-TTR	1	656.36	656.31	3.38	8.04	(11.37)	-1.7%
	2	650.94	668.70	3.42	8.64	(29.82)	-4.6%
	3	657.11	666.45	3.35	7.47	(20.16)	-3.1%
	4	650.99	670.19	3.63	6.77	(29.59)	-4.5%
ROH-RBP-muTTR	1	602.46	619.32	2.77	5.59	(25.21)	-4.2%
	2	598.84	620.44	3.22	4.95	(29.77)	-5.0%
	3	611.35	616.41	3.03	5.52	(13.61)	-2.2%
	4	606.02	614.02	2.68	5.15	(15.83)	-2.6%

bioRxiv preprint doi: https://doi.org/10.1101/2023.04.11.536348; this version posted April 12, 2023. The copyright holder for this preprint (which was not certified by peer review) is the author/funder, who has granted bioRxiv a license to display the preprint in perpetuity. It is made available under aCC-BY 4.0 International license.

Table S3: Mean apparent permeability of ^{3}H-ROH and ^{14}C-sucrose[2]

Sample	ROH Pe_{app} (10^{-6} cm/s)	Sucrose Pe_{app} (10^{-6} cm/s)
0.1 µM Free ROH	3.75 ± 0.39	0.43 ± 0.03
0.4 µM Free ROH	4.65 ± 0.43	0.48 ± 0.04
2 µM Free ROH	7.68 ± 0.53	0.58 ± 0.03
ROH-RBP	4.76 ± 0.19	0.69 ± 0.04
ROH-muRBP	5.44 ± 0.17	0.76 ± 0.06
ROH-RBP-TTR	6.56 ± 0.30	0.58 ± 0.03
ROH-RBP-muTTR	7.61 ± 0.13	0.55 ± 0.04

$$C_V = V_D \frac{S_{A,t}}{S_{D,t\,=\,60\,min}}$$ **Eq (S1)**

$$Pe_{app} = \frac{1}{A_F} m^{C_V}$$ **Eq (S2)**

[2] Pe_{app} is the mean apparent permeability (*N = 4 replicates*) of the BMEC monolayer and Transwell filter combined calculated by **Eq (S2)**, where A_F is the Transwell filter area and $m^{C_V}$is the fitted linear slope of the clearance volume (C_V) as a function of time. For samples displaying a lag-phase, slope was calculated only from the linear segment. C_V values were calculated by **Eq (S1)**, where V_D is the volume of the donor chamber and $S_{A,t}$ and $S_{D,t\,=\,60\,min}$ are the CPM signal in the acceptor chamber at time t and the CPM signal in the donor chamber at time $t = 60\;min$, respectively.

bioRxiv preprint doi: https://doi.org/10.1101/2023.04.11.536348; this version posted April 12, 2023. The copyright holder for this preprint (which was not certified by peer review) is the author/funder, who has granted bioRxiv a license to display the preprint in perpetuity. It is made available under aCC-BY 4.0 International license.

Table S4: Maximum percentage of fluid phase ROH accumulated by cells

Sample	Maximum percentage accumulated
0.1 μM Free ROH	16%
0.4 μM Free ROH	19%
2 μM Free ROH	28%
ROH-RBP	8%
ROH-muRBP	21%
ROH-RBP-TTR	9%
ROH-RBP-muTTR	8%

bioRxiv preprint doi: https://doi.org/10.1101/2023.04.11.536348; this version posted April 12, 2023. The copyright holder for this preprint (which was not certified by peer review) is the author/funder, who has granted bioRxiv a license to display the preprint in perpetuity. It is made available under aCC-BY 4.0 International license.

Table S5: Partitioning model fits for BMEC free ROH accumulation

Parameter	Eq 1. Fit	Eq 2. Fit
K_p (µM cell/ µM fluid)	131	99
k_1 (min^{-1})	0.021	0.027
$K_p{}^*$ (µM cell/ µM fluid)	N/A	90
$k_1{}^*$ (min^{-1})	N/A	0.014
t_{lag} (min)	N/A	0.1 µM Free ROH: N/A<br>0.4 µM Free ROH: 101<br>2 µM Free ROH: 7.8
$c_{cell}{}^*$ (µM)	N/A	36
χ^2	17,930	2,470
Residual Sum of Squares (RSS)	41,000	3,700
Aikake Information Criteria (AIC)	17,934	2,480

$$\frac{c_{cell}}{c_f} = K_p[1 - exp(-k_1 t)] \qquad \textbf{Eq. 1}$$

$$\frac{c_{cell}}{c_f} = K_p[1 - exp(-k_1 t)] + K_p{}^*\left[1 - exp\left(-k_1{}^*(t - t_{lag})\right)\right] \qquad \textbf{Eq. 2}$$

where

$$t_{lag} = \left. -ln\left[1 - \frac{c_{cell}{}^*}{K_p c_f}\right] \middle/ k_1 \right.$$

bioRxiv preprint doi: https://doi.org/10.1101/2023.04.11.536348; this version posted April 12, 2023. The copyright holder for this preprint (which was not certified by peer review) is the author/funder, who has granted bioRxiv a license to display the preprint in perpetuity. It is made available under aCC-BY 4.0 International license.

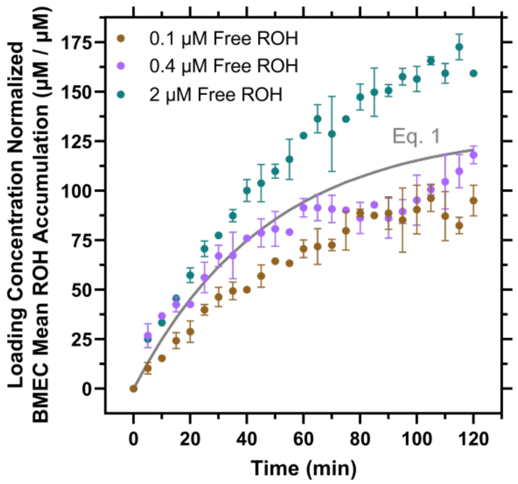

Figure S2: Mean ROH accumulation partitioning model. Data from Figure 4A are normalized by the ROH concentration in the medium and fit by a simple partitioning model (Eq. 1 in gray). If this model were correct, the data at all free ROH concentrations would collapse onto a single curve described by Eq. 1.

bioRxiv preprint doi: https://doi.org/10.1101/2023.04.11.536348; this version posted April 12, 2023. The copyright holder for this preprint (which was not certified by peer review) is the author/funder, who has granted bioRxiv a license to display the preprint in perpetuity. It is made available under aCC-BY 4.0 International license.

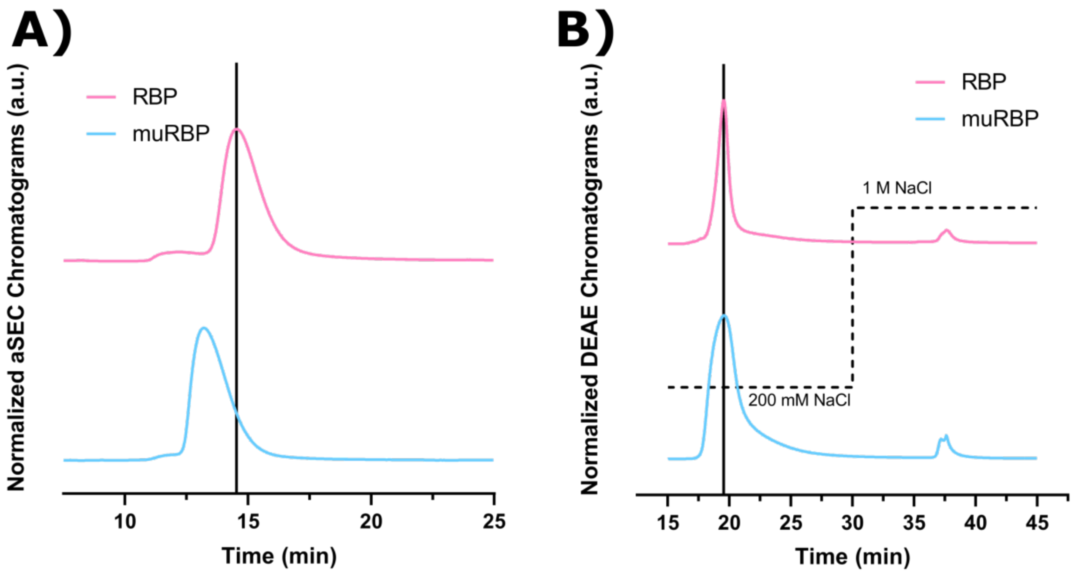

Figure S3: Normalized chromatographic analysis of wild-type RBP and L63R/L64S mutant RBP. Spectra of both wild-type RBP (RBP), adapted from (58), and L63R/L64S mutant RBP (muRBP) were normalized to their respective max absorbance signals at 280 nm and overlaid on the same plot. The vertical bars represent the retention time corresponding to the max signal for RBP. **A)** ~ 15 µg of either RBP or muRBP in PBS (pH 7.4) was injected onto a TOSOH TSKgel BioAssist G2SWxl analytical size exclusion column operating at a flow rate of 1.0 mL/min of PBS (pH 7.4). **B)** RBP and muRBP were concentrated and buffer exchanged into AEX Buffer A (25 mM Tris, 1 mM EDTA, pH 8.0). The protein samples were filtered through a 0.22 µm filter (Millipore) and slowly applied by syringe to a GE HiScreen diethylaminoethyl (DEAE) column pre-equilibrated with AEX Buffer A. The sample was allowed to adsorb for 10 minutes, then re-equilibrated for 10 minutes with AEX Buffer A at a flow rate of 1.0 mL/min. A step salt gradient (dotted line) was applied by mixing AEX Buffer A with high salt AEX Buffer B (25 mM Tris, 1 M NaCl, 1 mM EDTA, pH 8.0) at 1.0 mL/min.
